# MAdCAM-1-Mediated Intestinal Lymphocyte Homing Is Critical for the Development of Active Experimental Autoimmune Encephalomyelitis

**DOI:** 10.3389/fimmu.2019.00903

**Published:** 2019-04-26

**Authors:** Kristina Kuhbandner, Anna Hammer, Stefanie Haase, Elisa Terbrack, Alana Hoffmann, Angela Schippers, Norbert Wagner, Rehana Z. Hussain, William A. Miller-Little, Andrew Y. Koh, Joshua S. Stoolman, Benjamin M. Segal, Ralf A. Linker, Olaf Stüve

**Affiliations:** ^1^Department of Neurology, University Hospital Erlangen, Friedrich-Alexander-University Erlangen-Nuremberg, Erlangen, Germany; ^2^Department of Molecular Neurology, University Hospital Erlangen, Friedrich-Alexander-University Erlangen-Nuremberg, Erlangen, Germany; ^3^Department of Pediatrics, University Hospital RWTH Aachen, Aachen, Germany; ^4^Department of Neurology and Neurotherapeutics, University of Texas Southwestern Medical Center, Dallas, TX, United States; ^5^Department of Pediatrics, Microbiology, Simmons Comprehensive Cancer Center, University of Texas Southwestern Medical Center, Dallas, TX, United States; ^6^Department of Neurology, University of Michigan School of Medicine, Ann Arbor, MI, United States; ^7^Department of Neurology, University of Regensburg, Regensburg, Germany; ^8^Neurology Section, VA North Texas Health Care System, Dallas, TX, United States

**Keywords:** MAdCAM-1, lymphocyte homing, intestine, EAE/MS, neuroinflammation

## Abstract

Lymphocyte homing into the intestine is mediated by binding of leukocytes to mucosal addressin cell adhesion molecule 1 (MAdCAM-1), expressed on endothelial cells. Currently, the immune system of the gut is considered a major modulator not only of inflammatory bowel disease, but also of extra-intestinal autoimmune disorders, including multiple sclerosis (MS). Despite intense research in this field, the exact role of the intestine in the pathogenesis of (neuro-)inflammatory disease conditions remains to be clarified. This prompted us to investigate the role of MAdCAM-1 in immunological processes in the intestine during T cell-mediated autoimmunity of the central nervous system (CNS). Using the experimental autoimmune encephalomyelitis model of MS, we show that MAdCAM-1-deficient (MAdCAM-1-KO) mice are less susceptible to actively MOG_35−55_-induced disease. Protection from disease was accompanied by decreased numbers of immune cells in the lamina propria and Peyer's patches as well as reduced immune cell infiltration into the spinal cord. MOG_35−55_-recall responses were intact in other secondary lymphoid organs of MAdCAM-1-KO mice. The composition of specific bacterial groups within the microbiome did not differ between MAdCAM-1-KO mice and controls, while MAdCAM-1-deficiency severely impaired migration of MOG_35−55_-activated lymphocytes to the gut. Our data indicate a critical role of MAdCAM-1 in the development of CNS inflammation by regulating lymphocyte homing to the intestine, and may suggest a role for the intestinal tract in educating lymphocytes to become encephalitogenic.

## Introduction

Lymphocyte circulation and migration is crucial for the maintenance of immunological surveillance and is regulated by the interaction of immune competent cells with high endothelial venules (1). Various adhesion molecules and homing receptors are involved in these processes. For example, recruitment of lymphocytes into the gut mucosa is mediated by binding of cellular α4β7-integrin to mucosal addressin cell adhesion molecule 1 (MAdCAM-1) (2). This molecule is constitutively expressed on high endothelial venules of Peyer's patches (PP) as well as mesenteric lymph nodes, post-capillary venules of the intestinal lamina propria, the lactating mammary gland and in sinus-lining cells in the spleen (3, 4). Furthermore, Schippers et al. reported a central role of MAdCAM-1 in the formation of lymphoid tissue in the intestine, particularly during postnatal development, and in migration of immune cells to the lamina propria (5, 6). Interestingly, MAdCAM-1 was also found to be upregulated on inflamed venules in chronic inflammatory diseases, for example in the intestine in colitis and in choroid plexus epithelium during experimental autoimmune encephalomyelitis (EAE), an animal model of multiple sclerosis (MS) (7–9). Blockade of MAdCAM-1 by anti-MAdCAM-1-antibody showed very modest beneficial effects in different models of experimental colitis as well as in some phases of progressive, non-remitting EAE (10–13).

Various inflammatory diseases, including Crohn's disease and ulcerative colitis are organ-specific and restricted to the gut, and are likely caused by dysregulation of the intestinal immune system. However, there is evidence that gut immunity is also involved in the pathogenesis of extra-intestinal autoimmune disorders such as rheumatoid arthritis or MS [reviewed by Kamada et al. (14)]. Despite extensive research in this field, many questions regarding lymphocyte migration, especially in pathogenic conditions, and its molecular basis remain to be clarified.

Therefore, in the present study, we focus on the role of MAdCAM-1 in the development of T cell-mediated autoimmunity by inducing active MOG_35−55_ –EAE in MAdCAM-1-KO mice.

We show that MAdCAM-1-deficiency ameliorated the disease course in active EAE accompanied by significantly reduced lymphocyte recruitment to the intestinal lamina propria and infiltration of immune cells in the central nervous system (CNS). In summary, our data suggest a MAdCAM-1-associated modulation of CNS autoimmunity within the intestinal tract and add new insight into a functional role of the intestine in neuroinflammation.

## Materials and Methods

### Mice

MAdCAM-1-KO mice were a kind gift from Dr. Schippers, University hospital Aachen, Aachen, Germany. MAdCAM-1-KO mice and control littermates were backcrossed on the C57BL/6 background and were obtained from an in-house-breed at the local animal care facility at the University Hospital Aachen. Mice were housed at the Franz-Penzoldt-Zentrum, the local animal care facility of the University of Erlangen-Nürnberg under a 12-h day-night cycle and standardized environment or purchased from Charles River (Sulzfeld, Germany). All experiments were performed in accordance with the German laws of animal protection and were approved by local ethics committees (Government of Unterfranken, Bavaria, Germany, ref. # 55.2-2532-2-451). For additional experiments, mice were bred and maintained in a pathogen free mouse colony at the University of Texas Southwestern Medical Center (UTSW) in accordance to the guidelines set forth by the National Institute of Health and our institution. All experiments pertaining to these animals were approved by the UTSW Institutional Animal Care and Use Committee (IACUC).

### Induction of Active EAE

Induction of EAE in MAdCAM-1-KO mice and littermate controls was performed as previously described (15, 16). Briefly, 11–16 week old mice were anesthetized and subcutaneously injected with 200 μg MOG_35−55_ and 200 μg CFA. Pertussis toxin (200 ng/mouse) was applied intraperitoneally (i.p.) on days 0 and 2 post immunization (p.i.). Daily clinical evaluation was performed via a 5-point scale. For disease course, only mice showing clinical symptoms were included. EAE experiments with male and female MAdCAM-1-KO mice showed no sex differences regarding disease incidence and severity (data not shown).

For anti-MAdCAM-1-antibody experiments performed at UT Southwestern, individual animals were observed daily based on the EAE clinical scoring system as follows: 0 = no clinical disease, 1 = loss of tail tone, 2 = mild paraparesis, 3 = paraplegia, 4 = hind limb and forelimb paralysis, 5 = moribund or death.

### Therapeutic Antibodies

Mice were treated with 200 μg/100 μl of anti-MAdCAM-1 monoclonal antibody (Biolegend, clone MECA-367) intravenously (i.v.) on day 5 post immunization, and intraperitoneally on days 7, 10, 12, and 15 post immunization. Control mice received an equal volume of phosphate-buffered saline (PBS) via the same route at the same time points. This anti-MAdCAM-1 mAb blocks MAdCAM-1 *in vitro* and *in vivo*, and was shown by other investigators to ameliorate gastrointestinal inflammation in animal models (17).

### Transfer of e450—Labeled Splenocytes

EAE was induced in 11–13 week old donor mice as described before. After 8 days, splenocytes were isolated and labeled with eFluor^Ⓡ^450 proliferation dye (10 μM, eBioscience) according to the manufacturer's instructions. 15 × 10^6^ labeled cells were injected i.v. in MAdCAM-1-KO and littermate recipient mice. Recipients were immunized with MOG_35−55_ peptide and pertussis toxin 4 days prior to splenocyte injection. 4 days after cell transfer, different organs of these mice were analyzed for the presence of e450^+^ cells by flow cytometry.

### Isolation of Splenocytes

Spleens of mice were removed 10 days p.i. and disrupted with a 5 ml glass homogenizer. The tissue was then filtered through a 100 μm cell strainer followed by erythrocyte lysis. Subsequently, cells were processed for flow cytometric analysis (see section Flow Cytometry).

### Isolation of CNS Infiltrating Cells

Spinal cord was removed at the maximum of disease and disrupted with a 5 ml glass homogenizer. Isolated cells were purified by a three-step density gradient using 30%, 45% and 70% isotonic Percoll™ (GE Healthcare). After centrifugation without brake (20 min, 800 g, 18°C), CNS infiltrating lymphocytes were harvested from the interphases, washed with cold PBS and processed for *ex vivo* flow cytometry analysis (see section Flow Cytometry).

### Isolation of Cells From the Small Intestine

At different time points of EAE, single cell suspensions from lamina propria and the intestinal epithelium were obtained using the Lamina Propria Dissociation Kit (Miltenyi, Bergisch Gladbach, Germany). To obtain single cell suspensions of intestinal epithelium, gut pieces were de-epithelialized in a predigestion solution containing EDTA and DTT. Cell suspensions from the lamina propria were obtained by enzymatic and mechanic dissociation of the intestinal pieces. Working steps were done according to the manufacturer's protocol. After isolation, cells were extra- and intracellularly stained for *ex vivo* flow cytometry analysis (see section Flow Cytometry).

### Flow Cytometry

*Ex vivo*-obtained CNS and splenic lymphocytes as well as cells isolated from the small intestine, blood, PP and other lymphatic organs were analyzed by staining for extra- and intracellular markers. Dead cells were excluded using the fixable viability dye eFluor^Ⓡ^780 (0.2 μl/test, eBioscience). Non-specific Fc-mediated interactions were blocked by addition of 0.5 μl αCD16/32 (93, eBioscience). For surface staining, cells were treated with the following fluorochrome-conjugated antibodies for 30 min in PBS: αB220-BV510 (RA3-6 B2, Biolegend), αB220-APC (RA3-6 B2, Biolegend), αB220-FITC (RA3-6 B2, Biolegend), αCD3-BV421 (17A2, BD Biosciences), αCD4-FITC (RM4-5, eBioscience), αCD4-APC (RM4-5, Biolegend), αCD8a-BV510 (53-6.7, Biolegend), αCD8a-PerCP (53-6.7, Biolegend), αCD11b-APC (M1/70, Biolegend), αCD11b-PE (M1/70, Biolegend), αCD11c-PE/Cy7 (N418, Biolegend), αCD25-PECy5 (PC61, Biolegend), αCD45-FITC (30-F11, Biolegend).

For intracellular cytokine staining, cells were stimulated for 4 h with ionomycin (1 μM, Sigma-Aldrich) and PMA (50 ng/ml, Sigma-Aldrich) in the presence of monensin (2 μM, eBioscience). After fixation with either 1% paraformaldehyde (PFA) and permeabilization with saponin buffer or with Foxp3/Transcription Factor Staining Buffer Set (eBioscience), intracellular cytokines were stained with the following fluorochrome-conjugated antibodies for 30–45 min: αFoxp3-PE (FJK-16s, eBioscience), αIFNγ-PE/Cy7 (XMG1.2, BD Pharmingen), and αIL-17A-PE (eBio17B7, eBioscience), and αTNFα-APC (MP6-XT22, Biolegend). Cells were measured with a flow cytometer (FACSCantoII or FACSFortessa, BD Biosciences) and FACS data were analyzed using FlowJo software (BD Biosciences). At UT Southwestern, flow cytometry was performed with a FACSAria II flow cytometer (BD Biosciences), equipped with Diva acquisition software (BD Biosciences). FlowJo (BD Biosciences) software was also utilized for some data analyses.

### *In vitro* MOG Restimulation Assay

Splenocytes from EAE mice were isolated on day 10 p.i., seeded at a density of 3 × 10^6^ cells/ml and stimulated with MOG_35−55_ (20 μg/ml) or Concanavalin A (Con A) (1.25 μg/ml). After 48 h, supernatants were harvested and analyzed for cytokines (see section Enzyme-Linked Immunosorbent Assay).

### Culture of Intraepithelial Intestinal Immune Cells, Lamina Propria Cells, and Splenocytes

Intraepithelial intestinal immune cells, lamina propria cells and splenocytes isolated as described above were seeded at a density of 1 × 10^6^ cells/ml and cultured for 2 days with plate-bound αCD3 (2 μg/ml, 145-2C11, BD Pharmingen) and soluble αCD28 (2 μg/ml, 37.51, BD Pharmingen). After supernatant collection, cytokines were measured by enzyme-linked immunosorbent assay (ELISA) (see section Enzyme-Linked Immunosorbent Assay).

### Enzyme-Linked Immunosorbent Assay (ELISA)

Concentrations of IL-17A and IFN-γ in cell culture supernatants were measured by ELISA (DuoSet ELISA kits, R&D) according to the manufacturer's instructions.

### Immunohistochemistry and Tissue Staining

Spinal cords and spleens of EAE mice were removed following perfusion with 4% (wt/vol) PFA and post-fixed for 2–3 h. After embedding in paraffin, 4 μm thin sections were prepared by using a microtome. For immunohistochemistry, αCD3 (1:200, MCA1477, Bio-Rad) and αMac-3 (1:200, M3/84, BD Pharmingen) antibodies were used to detect immune cells. Luxol Fast Blue staining was performed for evaluation of demyelination and Bielschowksy silver impregnation for axonal integrity/damage. Quantification of axonal preservation, cellular infiltrates, and degree of demyelination was performed in a blinded fashion on 9 independent spinal cord sections per mouse. Cellular infiltrates were quantified per square millimeter of white matter by overlaying a stereological grid onto sections and demyelinated areas were determined semi-automatically by CellP Software (Olympus). Six visual fields of the cervical, thoracic, and lumbar spinal cord were used for quantification of axonal preservation counted on a 100 μm diameter grid.

### Real-Time PCR

MAdCAM-1 gene expression was analyzed by real-time PCR. Total RNA was isolated using the PEQgold HP total RNA kit (peqlab). RNA yield was quantified by absorbance measurements at 260 nm. Total RNA (500–1,000 ng per reaction) was used to reversely transcribe RNA into cDNA employing the QuantiTect Reverse Transcription Kit (Qiagen). PCR reactions were performed at a 5 μl scale with a qTower real-time PCR System (Analytik Jena). Relative quantification was performed by the ΔΔCT method, normalizing target gene expression on actb/β-Actin as housekeeping gene. The following TaqMan real-time PCR assays from Thermo Fisher Scientific were used: actb (β-Actin) Mm00607939_s1 and Madcam1 Mm00522088_m1.

For microbiota analysis, bacterial loads were quantified by qPCR analysis (SsoAdvanced SYBR Green Supermix, Bio-Rad) of microbial gDNA using universal 16S rRNA gene internal transcribed spacer (ITS1-2) primers. The abundance of specific bacterial groups was determined by qPCR using group-specific 16S rRNA gene primers. Bacterial abundance was determined using standard curves constructed with reference to cloned DNA corresponding to a short segment of the 16s rRNA that was amplified using the following conserved specific primers: Eubacteria (all bacteria) ACTCCTACGGGAGGCAGCAGT (forward primer)–TACCGCGGCTGCTGGC (reverse primer); Bacteroides GGTTCTGAGAGGAGGTCCC (forward primer)–GCTGCCTCCCGTAGGAGT (reverse primer); Mouse Intestinal Bacteroides (MIB) CCAGCAGCCGCGGTAATA (forward primer)–CGCATTCCGCATACTTCTC (reverse primer); Lactobacillus/Enterococcus Group (LACT) AGCAGTAGGGAATCTTCCA (forward primer)–CACCGCTACACATGGAG (reverse primer); Eubacterium rectale/Clostridium coccoides group (EREC) ACTCCTACGGGAGGCAGC (forward primer)–GCTTCTTAGTCAGGTACCGTCAT (reverse primer); Clostridium leptum group (CLEPT) GCACAAGCAGTGGAGT (forward primer)–CTTCCTCCGTTTTGTCAA (reverse primer); Enterobacteriaceae (ENTERO) GTGCCAGCMGCCGCGGTAA (forward primer)–GCCTCAAGGGCACAACCTCCAAG (reverse primer); Segmented filamentous bacteria (SFB) GACGCTGAGGCATGAGAGCAT (forward primer)–GACGGCACGGATTGTTATTCA(reverse primer).

### Statistical Analysis

Statistical testing was performed using GraphPad Prism (GraphPad Software Inc., San Diego, CA, USA). All *in vitro* and *ex vivo* data were analyzed by one-/two-way ANOVA followed by Tukey's posttest or unpaired *t*-test (unless otherwise indicated). EAE data were analyzed either by Mann–Whitney *U*-test, unpaired *t*-test (for testing of single time points) or logrank test (for disease incidence analysis). Data are presented as mean ± SEM; ^*^p < 0.05, ^**^p < 0.01, or ^***^p < 0.001 were considered to be statistically significant.

## Results

### MAdCAM-1 Is Abundantly Expressed in the Intestinal Tract and Spleen of Wild Type Mice and Is Differentially Regulated in CNS During Active EAE

**Figure 1:**
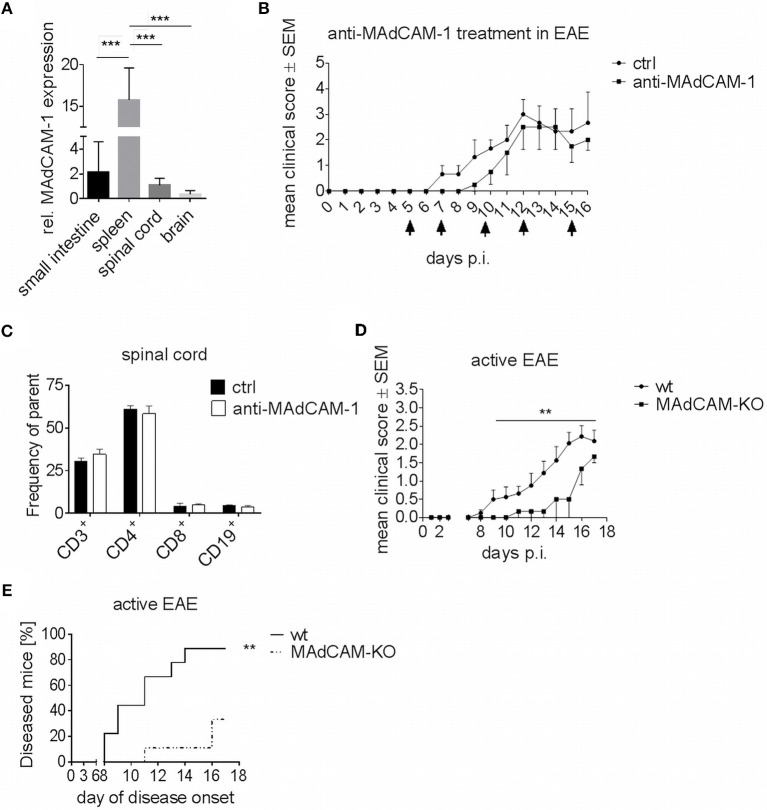
MAdCAM-1-deficiency reduces incidence of active EAE in C57BL/6 mice and ameliorates clinical symptoms. **(A)** MAdCAM-1 gene expression relative to naive small intestine in spleen, spinal cord and brain at the maximum of EAE in C57BL/6 mice (*n* = 8–13 mice per tissue, ^***^p < 0.001). **(B)** Clinical course of MOG_35−55_-EAE in mice treated with anti-MAdCAM-1-antibody or PBS (ctrl) based on a 5-point score scale (one representative out of two experiments is shown, controls: *n* = 3, treated mice: *n* = 4, only mice exhibiting EAE symptoms are shown, arrows indicate days of treatment). **(C)** *Ex vivo* flow cytometry analysis of leukocytes in the spinal cord after treatment with anti-MAdCAM-1-antibody or PBS (ctrl) on day 16 of EAE (*n* = 4 per group, average scores at the day of euthanasia: ctrl: 2.0, anti-MAdCAM-1: 2.0). **(D)** Clinical course of MOG_35−55_-EAE in wildtype (wt) and MAdCAM-KO mice. Data are shown on a 5-point score scale (wt: *n* = 8, MAdCAM-KO: *n* = 3, only mice exhibiting EAE symptoms are shown, data pooled from two independent experiments, ^**^p < 0.05). **(E)** Analysis of EAE incidence in wt and MAdCAM-KO mice (*n* = 9 per group, data pooled from two independent experiments, ^**^p < 0.01).

Gene expression analysis revealed that MAdCAM-1 is abundantly expressed in the small intestine, but also in spleen and CNS of C57BL/6 wild type (wt) mice at the maximum of EAE (Figure 1A). In accordance with previous studies showing upregulation of MAdCAM-1 in inflammatory diseases, we observed an increased MAdCAM-1 mRNA expression in the spinal cord and brain during active EAE induced by immunization with MOG_35−55_ peptide with a peak at maximum disease activity (data not shown).

### Blockade of MAdCAM-1 Does Not Significantly Impact on the Disease Course in Active EAE

To closer study the role of MAdCAM-1 in neuroinflammation, we investigated the effect of the absence of this molecule in active EAE. In a first approach, we used a monoclonal anti-MAdCAM-1-antibody to specifically block the interaction of MAdCAM-1 with α4β7-integrins on lymphocytes. C57BL/6 wt mice were treated with anti-MAdCAM-1-antibody starting 5 days after immunization with MOG_35−55_ peptide. As compared to sham-treated controls, mice treated with anti-MAdCAM-1-antibody demonstrated no significant difference at any phase of the disease (Figure 1B). Finally, there was no difference in the frequency of CD3^+^, CD4^+^, and CD8^+^ T cells or CD19^+^ B cells in the brain (data not shown) and spinal cords of mice treated with anti-MAdCAM-1-antibody as compared to controls (Figure 1C).

### MAdCAM-1-Deficiency Significantly Ameliorates Active EAE

Molecule-specific antagonism by therapeutic monoclonal antibodies has limitations due to inadequate pharmacokinetics and limited tissue distribution. Therefore, we extended our initial findings beyond the pharmacological approach by examining the effect of genetic MAdCAM-1-deficiency in neuroinflammation. Active EAE was induced in MAdCAM-1-KO mice and wt littermates with physiological MAdCAM-1 expression. In contrast to anti-MAdCAM-1 therapy, MAdCAM-1-deficiency substantially ameliorated the clinical disease course and significantly lowered disease incidence compared to the control group. After an observation period of 17 days, 89% of littermate controls developed EAE, while only 33% of MAdCAM-1-KO mice showed signs of disease (Figures 1D,E). The mortality rate did not differ between both groups.

### MAdCAM-1-Deficiency Impedes Immune Cell Infiltration in the Spinal Cord After Active EAE Induction

**Figure 2:**
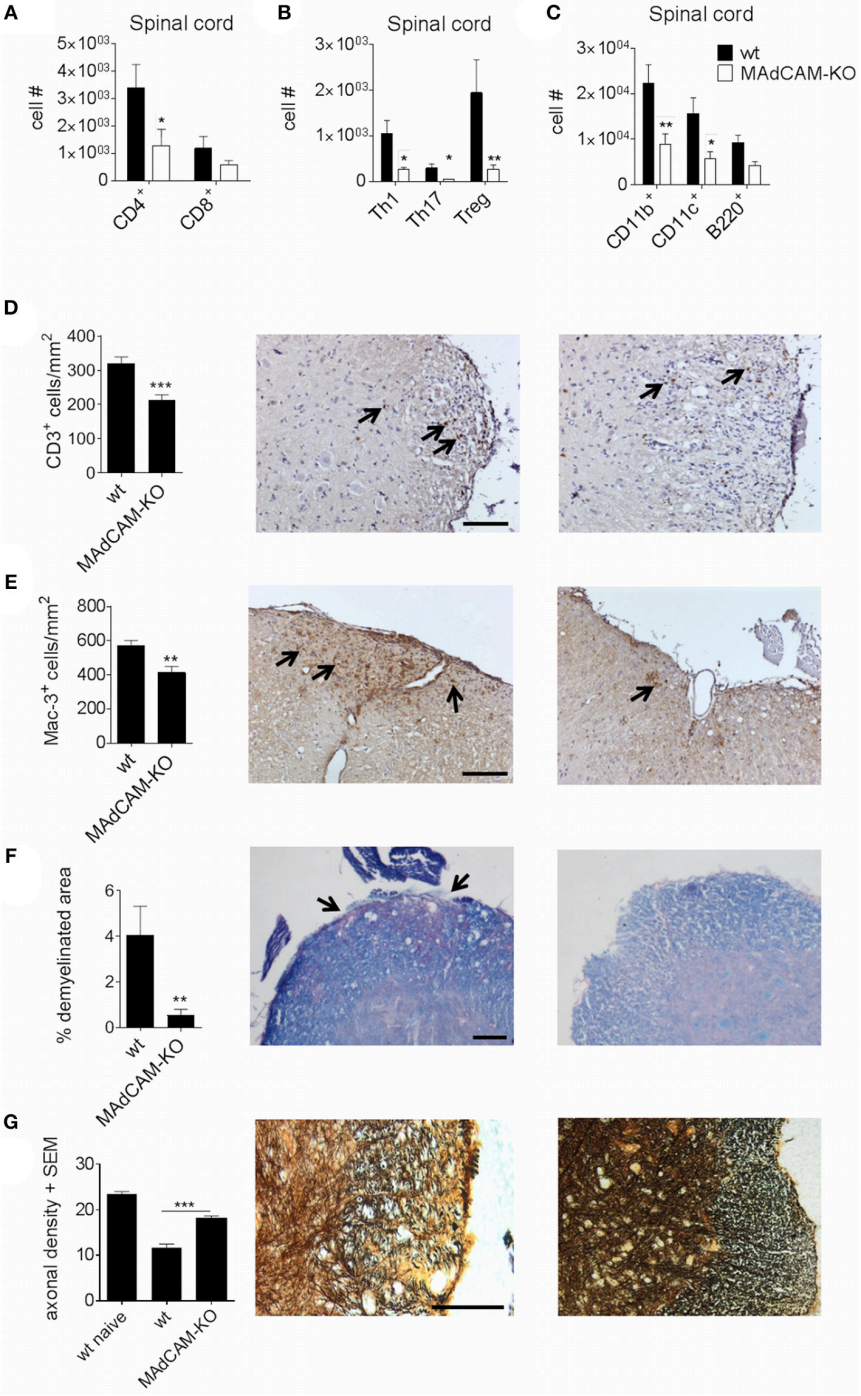
MAdCAM-1-deficiency hampers immune cell infiltration in the spinal cord after active EAE induction. **(A–C)** *Ex vivo* flow cytometry analysis of infiltrating immune cells in the spinal cord of wt and MAdCAM-KO mice at the maximum of disease (*n* = 9 mice per group, average score at the day of euthanasia: wt: 2.6, MAdCAM-KO: 1.1; ^*^p < 0.05 ^**^p < 0.01). **(D–G)** Histological analyses of T cell **(D)** and macrophage **(E)** infiltration, demyelination **(F)** in the spinal cord of wt and MAdCAM-KO mice, and axonal densities **(G)** in the spinal cord of naïve wt, wt, and MAdCAM-KO mice (*n* = 4 mice per group, ^**^p < 0.01, ^***^p < 0.001). Perfusion and spinal cord removal for histology was done on day 23 p.i. of MOG_35−55_-EAE. Representative images from lumbar spinal cord cross sections are shown (arrows indicate CD3^+^ cells, Mac-3^+^ cells or demyelinated area, scale bar 100 μm for each image).

To further examine the effect of MAdCAM-1 on cellular events that drive CNS autoimmunity, we analyzed CNS infiltrating immune cells at the maximum of active EAE by *ex vivo* flow cytometry. The actively-induced MOG_35−55_-EAE model localizes primarily to the distal spinal cord and is initiated by antigen-specific CD4^+^ T helper (Th) cells, and perpetuated by CD4^+^ T cells, CD8^+^ T cells, and pro-inflammatory myeloid cells. The infiltration of CD4^+^ and CD8^+^ T cells in the spinal cord of MAdCAM-1-KO mice was reduced compared to littermate controls (Figure 2A). Within the T cell compartment, the number of Th1 and Th17 cells as well as regulatory T cells (Treg) was significantly decreased (Figure 2B). Also, the number of CD11b^+^ tissue macrophages and CD11c^+^ dendritic cells in the spinal cord was diminished during EAE (Figure 2C). Immunohistological analysis confirmed these findings showing reduced numbers of CD3^+^ T cells and Mac-3^+^ mononuclear phagocytes in the spinal cord at the maximum of disease (Figures 2D,E). Additionally, the extent of demyelination, as assessed by Luxol fast blue staining, was significantly lower and axonal densities, determined by Bielschowsky silver impregnation, were higher in MAdCAM-1-KO mice (Figures 2F,G).

### MAdCAM-1-Deficiency Does Not Affect Immune Cell Frequencies in the Spleen

**Figure 3:**
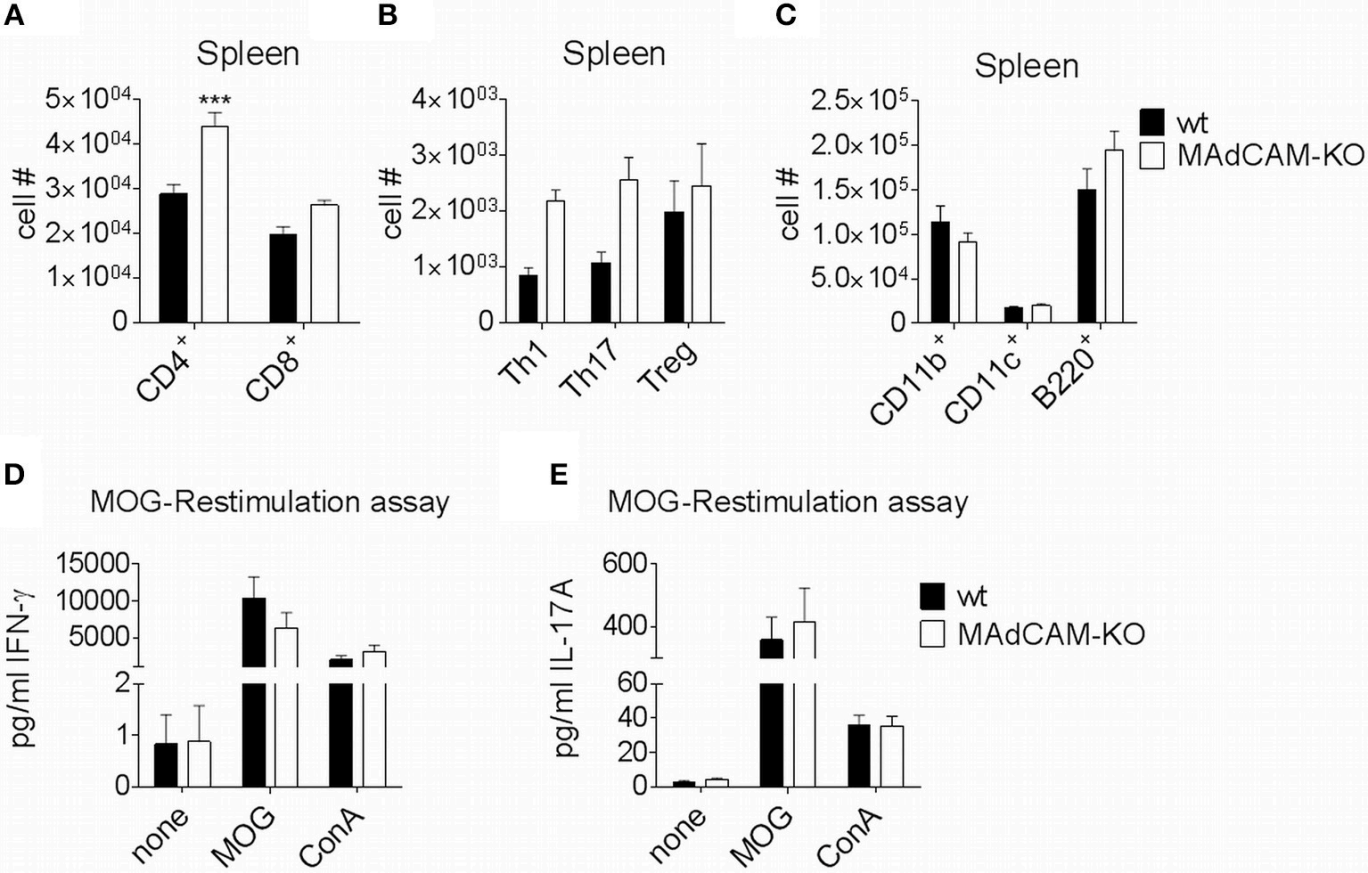
MAdCAM-1-deficiency does not diminish immune cell numbers in the spleen. **(A–C)** *Ex vivo* flow cytometry analysis of immune cell numbers in the spleen of wt and MAdCAM-KO mice on day 10 of MOG_35−55_-EAE (*n* = 10 mice per group, ^***^p < 0.001). **(D,E)** Cytokine measurement in cultures after *ex vivo* restimulation with MOG_35−55_ or ConA (splenocytes harvested on day 10 p.i. of MOG_35−55_-EAE, *n* = 10 per group). MOG, myelin oligodendrocyte glycoprotein; ConA, Concanavalin A.

The effect of MAdCAM-1-deficiency on cellular infiltration of the spinal cord during active EAE prompted us to study its effect on secondary lymphoid organs relevant for EAE pathogenesis. Specifically, we performed *ex vivo* immunophenotyping of splenocytes 10 days after EAE induction. The number of CD4^+^ T cells in the spleen of MAdCAM-1-KO mice was significantly higher and numbers of Th1, Th17 and Treg cells was slightly increased compared to controls, whereas the numbers of CD11b^+^ tissue macrophages, CD11c^+^ dendritic cells, and B220^+^ cells were not altered (Figures 3A–C). Similar results were obtained for splenocytes harvested at the maximum of disease (data not shown). Restimulation assays with isolated splenocytes on day 10 p.i. revealed no differences in the production of IFN-γ and IL-17A between MAdCAM-1-KO and control mice (Figures 3D,E), essentially ruling out a systemic defect in T helper cell responses.

### MAdCAM-1-KO Mice Display Reduced Numbers of Immune Cells in the Lamina Propria and Peyer's Patches After EAE Induction

**Figure 4:**
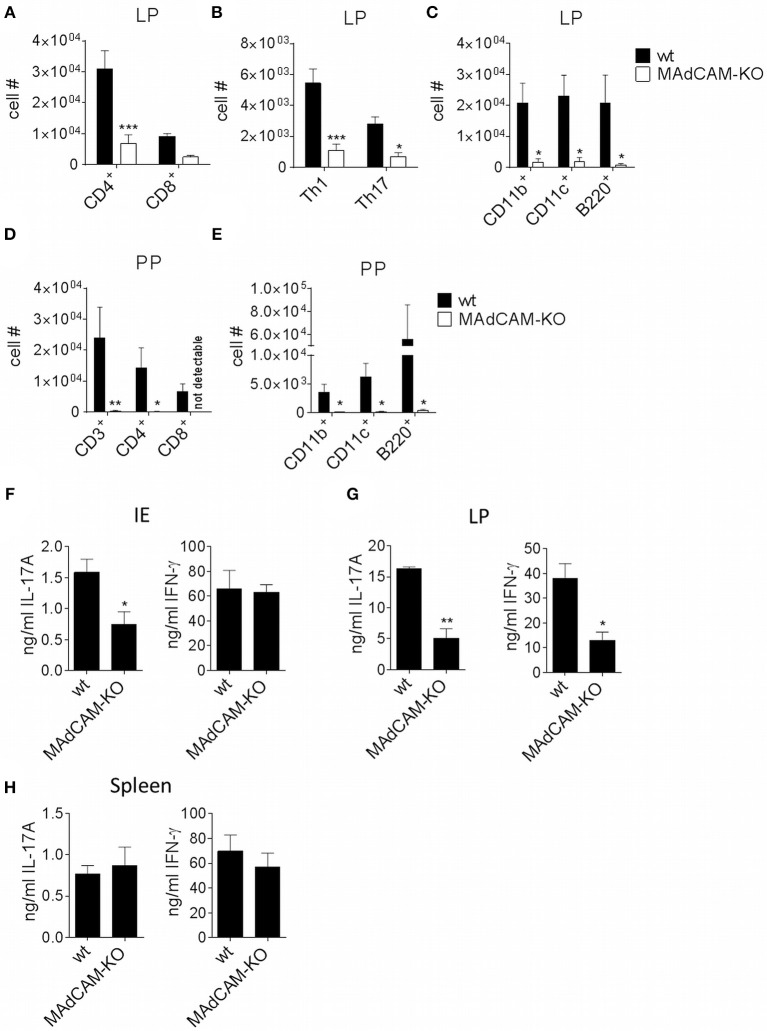
MAdCAM-KO mice display reduced numbers of immune cells in the lamina propria and Peyer's patches after EAE induction. **(A–E)** Flow cytometry analysis of cells isolated from the lamina propria **(A–C)** or PP **(D,E)** of wt and MAdCAM-KO mice on day 3 after EAE induction (*n* = 8 mice per group, ^*^p < 0.05, ^**^p < 0.01, ^***^p < 0.001). **(F–H)** Cytokine measurement in cultures of IE cells **(F)**, LP cells **(G)** or splenocytes **(H)** after *ex vivo* stimulation with αCD3 and αCD28 for 48 h (cells harvested on day 3 p.i. of MOG_35−55_-EAE, *n* = 4 per group, one representative out of two independent experiments is shown, ^*^p < 0.05, ^**^p < 0.01). IE, intestinal epithelium; LP, lamina propria; PP, Peyer's patches.

As MAdCAM-1 is predominantly expressed in the small intestine and plays an essential role in immune cell infiltration in the gut, we studied the impact of MAdCAM-1-deficiency on immune cell frequencies in the small intestine during active EAE. *Ex vivo* flow cytometry analysis of the small intestine 3 days after EAE induction showed a significantly decreased absolute number of immune cells in the lamina propria of MAdCAM-1-KO mice. Besides a strong reduction in Th1, Th17 and Treg cells, CD11b^+^, CD11c^+^ and B220^+^ cells were also diminished in MAdCAM-1-KO mice compared to littermate controls (Figures 4A–C). However, there were no differences in immune cell numbers in the intestinal epithelium. Consistent with the findings from Schippers et al. (5), the number of PP in the small intestine was significantly reduced in MAdCAM-1-KO mice (2.7 vs. 6.2 in control mice, *p* < 0.0001, *n* = 12 mice per group, data not shown). Moreover, the number of CD3^+^, CD4^+^, and CD8^+^ T cells as well as CD11b^+^, CD11c^+^, and B220^+^ antigen-presenting cells were reduced in PP (Figures 4D,E). Additionally, we measured cytokine secretion after *ex vivo* stimulation in culture supernatants of immune cells isolated from the intestinal epithelium, the lamina propria or the spleen 3 days p.i. (Figures 4F–H). Production of IL-17A and IFN-γ by lamina propria immune cells from MAdCAM-1-KO mice was markedly reduced, whereas in supernatants of intraepithelial immune cell cultures only IL-17A production was decreased compared to controls (Figures 4F,G). In accordance with restimulation assay data obtained from splenocytes analyzed 10 days after EAE induction, there was no difference in cytokine secretion in splenocyte culture supernatants (Figure 4H).

### The Composition of Specific Bacterial Groups Within the Microbiome of MAdCAM-1-KO Mice Is Within Normal Limits

**Figure 5:**
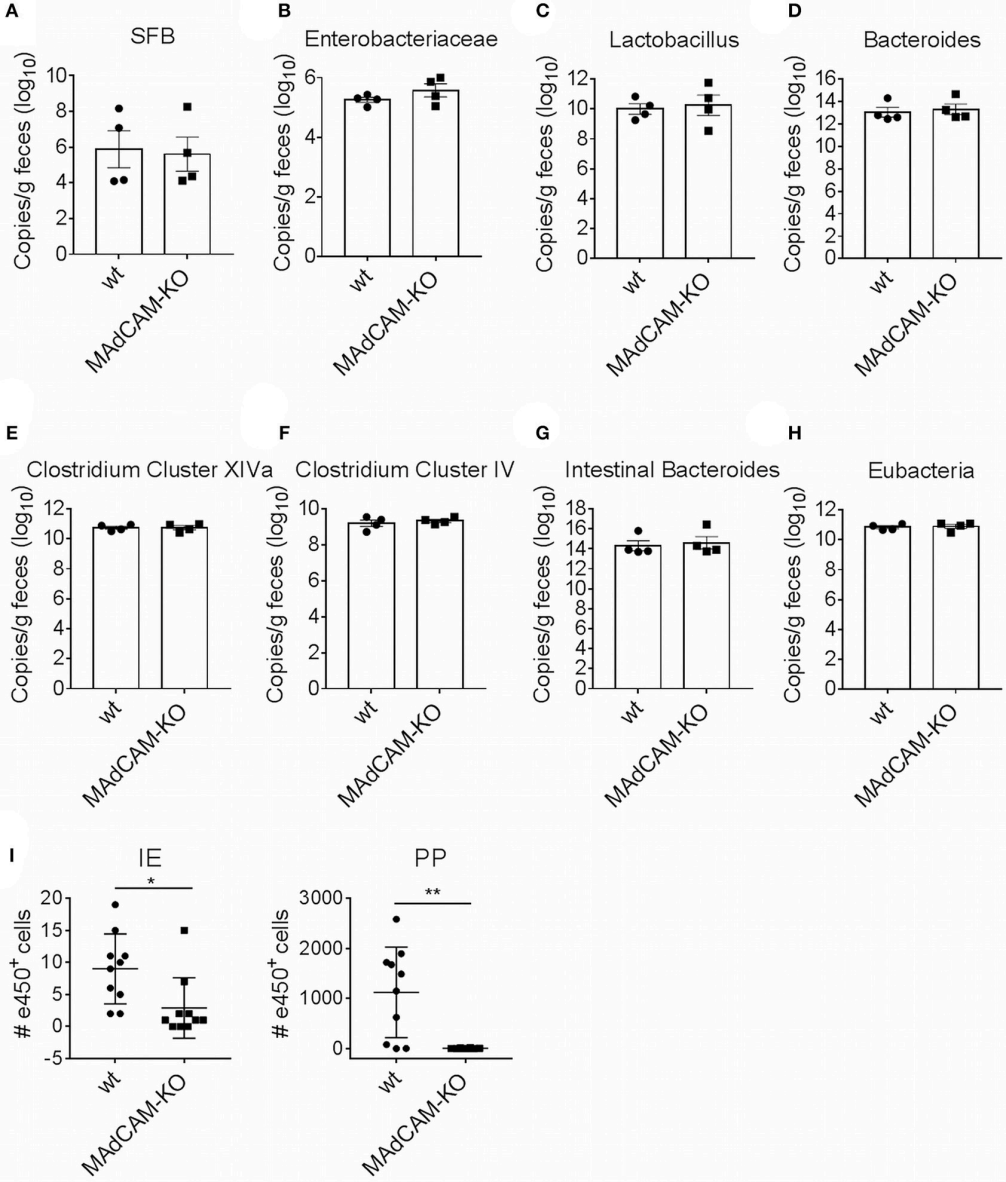
MAdCAM-1-deficiency does not alter microbiome composition in naive mice. **(A–H)** Quantification of bacterial load by qPCR-analysis for different microbe strains associated with neuroinflammatory processes (*n* = 4 per group, displayed in copies/g feces). **(I)** Number of e450^+^ cells in IE and PP 4 days after injection of e450-labeled splenocytes in recipient mice on day 4 of EAE (splenocytes isolated from donors 7 days after EAE induction, *n* = 8–10 mice per group, ^*^p < 0.05, ^**^p < 0.01). SFB, segmented filamentous bacteria; IE, intestinal epithelium; PP, Peyer's patches.

One possible explanation for observed immunological alterations in the gut may be a change in the composition of the microbiome. We thus performed microbiome analysis for common strains known to play a role in neuroinflammation, e.g., Bacteroides, Lactobacillus, Enterobacteriaceae and Segmented Filamentous Bacteria. No major differences in gene expression were observed between MAdCAM-1-KO and littermate controls (Figures 5A–H), suggesting that immunological effects of MAdCAM-1 in the gut are not governed by major changes in the microbiota.

### MAdCAM-1-Deficiency Reduces Access of Myelin-Activated Lymphocytes to the Intestinal Tract

To determine the potential role of MAdCAM-1 in the trafficking of myelin-activated lymphocytes during actively-induced EAE, we adoptively transferred e450-labeled splenocytes from donors immunized with MOG_35−55_ peptide. MAdCAM-1-KO and littermate recipients were immunized 4 days prior to injection of cells. Eight days after EAE induction, the numbers of e450^+^ cells in spleen, blood, inguinal and axillary as well as mesenteric lymph nodes determined by flow cytometry did not significantly differ between MAdCAM-1-KO mice and littermate controls (data not shown). However, significantly fewer e450^+^ cells were detected in the intraepithelial compartment and the PP of MAdCAM-1-KO mice compared to littermate controls (Figure 5I). This observation underlines the role of MAdCAM-1 in the access of myelin-activated lymphocytes to the intestinal tract.

## Discussion

Our data indicate that MAdCAM-1 is critically involved in the regulation of lymphocyte migration into the intestine and gut-associated lymphoid tissue during CNS autoimmunity. MAdCAM-1-deficiency significantly ameliorated EAE disease course and lowered disease incidence after active immunization with MOG_35−55_ peptide. This was accompanied by a considerable decrease in immune cell infiltration into the small intestine as well as into the spinal cord, but no significant quantitative or qualitative effects in the spleen. We thus propose a role for MAdCAM-1 in the acquisition of encephalitogenicity by T helper cell subsets in the intestinal tract after active induction of EAE.

In general, the complex process of lymphocyte migration is controlled by lymphocyte-endothelial cell recognition and requires the interaction of homing receptors expressed on lymphocytes with endothelial adhesion molecules. The differential expression of these cell surface molecules in different tissues facilitates an organ-specific regulation of lymphocyte trafficking. For example, lymphocyte homing to the gut is specifically regulated by binding of cellular integrin α4β7 to MAdCAM-1 (2).

MAdCAM-1 is known to be constitutively expressed on high endothelial venules of the gut and gut-associated lymphoid tissue (3). In kinetic gene expression analysis, we showed that MAdCAM-1 is expressed in the CNS of mice immunized with MOG_35−55_ peptide. This finding is well in line with previous studies showing increased expression of adhesion molecules in an inflammatory environment. For example, expression of MAdCAM-1 is upregulated in different animal models of colitis (8, 18, 19) as well as in EAE (9, 20). However, its role in lymphocyte migration into the CNS remains ambiguous and the discussion about the presence of MAdCAM-1 in the human brain is still ongoing (21–24). Only recently Döring et al. showed that TET-inducible MAdCAM-1 expression at the blood-brain barrier (BBB) neither influenced the clinical course nor the infiltration of immune cells into the CNS during active EAE (25). This supports the common notion that the recruitment of pathogenic T cells across the BBB is rather mediated by the interaction of α4β1-integrin with vascular adhesion molecule-1 (VCAM-1) and intercellular adhesion molecule-1 (ICAM-1), which are adhesion molecules with structural homology to MAdCAM-1 (26–29).

Much more is known about the specific role of MAdCAM-1 in the intestine. Here, it constitutes the main adhesion molecule involved in the specific recruitment of leukocytes to the gut. This knowledge is mainly derived from animal experiments using antibodies directed against MAdCAM-1 or its associated homing receptor α4β7 in naïve mice or in colitis models (2, 12, 13). Blockade of MAdCAM-1 in disease models, and therefore inhibition of immune cell infiltration to inflammatory sites, resulted in attenuated colitis. Furthermore, mice lacking MAdCAM-1 display alterations in size and cell composition of PP, reduction in the number of IgA-secreting plasma cells and an impaired intestinal IgA response (5).

Currently, the intestinal immune system, which comprises about 70% of the entire human immune system, is subject of intense research (30). Obvious correlations between intestinal autoimmune diseases such as Crohn's disease or ulcerative colitis and non-intestinal autoimmune disorders like MS have prompted researchers to closer investigate the involvement of intestinal inflammation in the development of autoimmunity (31, 32). Considering these points and the fact, that MAdCAM-1 plays a central role in the development of the intestinal immune system, we investigated the effect of MAdCAM-1 blockade and deficiency on intestinal immune cell composition in the EAE model. Treatment with an anti-MAdCAM-1-antibody did not show any significant effect during acute EAE. These data are consistent with previous experiments showing no substantial effects of anti-MAdCAM-1-antibody treatment in a model of chronic progressive, non-remitting EAE in the later disease stage (10). In contrast, active induction of MOG_35−55_–EAE in MAdCAM-1-KO mice resulted in an attenuated disease course and a significantly lower disease incidence compared to controls. The differences in the EAE course and disease incidence between these two models of MAdCAM-1-deficiency may be due to limitations of the therapeutic approach regarding inadequate pharmacokinetics and tissue distribution or due to the experimental setting as antibody-treatment was started 5 days after EAE induction. Considering that the intestinal tract may be an important player in the development of EAE, this timepoint is likely too late and therefore observed effects are only marginal.

Reflecting the clinical phenotype in MAdCAM-1-KO mice, immune cell infiltration into the spinal cord was reduced at the peak of EAE. However, we observed no alterations in peripheral immune responses as immune cell frequencies in the spleen were similar between MAdCAM-1-KO and littermate controls. Additionally, *in vitro* assays with re-stimulated splenocytes displayed no functional differences with respect to cytokine production. Consistent with the already known function of MAdCAM-1 in immune cell trafficking to the intestine, MAdCAM-1-deficiency significantly reduced the number of immune cells in the lamina propria and PP during EAE. These results imply that MAdCAM-1-deficiency does not impair systemic immunological processes and therefore, the differences in disease course observed in the absence of MAdCAM-1 might be based on the lack of T cell migration into the intestine. Migration experiments revealed reduced numbers of labeled splenocytes in the intraepithelial compartment and PP of MAdCAM-1-KO mice underlining the importance of MAdCAM-1 in cell migration to intestinal sites under (neuro-)inflammatory conditions.

In order to gain encephalitogenic properties and being able to enter the CNS, T cells have to go through multiple steps of activation by migrating to different organs. Flügel et al. showed that in a rat EAE model myelin basic protein-specific CD4^+^ T cells first migrate from parathymic lymph nodes to the blood and the spleen (33). Moreover, they determined the lung as specific tissue involved in autoimmune T cell reactivation in the preclinical phase of EAE (34). More recent studies also identified the intestine as an important site for immune cell priming, allocating the gut a critical role in development of EAE. For example it was shown, that encountering of immune cells with nutritional metabolites or microbiota regulates shaping of anti-inflammatory Tregs, but also pro-inflammatory Th17 cells (15, 35, 36). With respect to the suggested association between microbiota and the immunological processes in EAE, we also compared microbe composition between MAdCAM-1-KO and littermate control mice. qPCR analysis did not reveal apparent changes in the composition of the microbiome, perhaps arguing against a functional role of the microbiome in the relative disease resistance of actively-induced EAE in these mice. However, the qPCR that was employed has limitations in that it only amplifies gene copies of common bacterial species or families of species. Shotgun sequencing would be required to provide a comprehensive comparative microbiome analysis between MAdCAM-1-KO mice and controls.

Our data reinforce the concept that the intestine is critically involved in the development of autoimmune reactions and is an important site for immune cell priming during EAE. We suggest that inhibition of immune cell trafficking into the intestine by MAdCAM-1 knock out prevents priming and activation of T cells which is crucial for the initiation of autoimmune responses in the CNS in this model of neuroinflammation.

## Ethics Statement

This study was carried out in accordance with the recommendations of the German laws of animal protection, Government of Unterfranken, Bavaria, Germany and in accordance with the recommendations by the National Institute of Health, UTSW Institutional Animal Care and Use Committee (IAUAC).

## Author Contributions

KK, AnH, ET, SH, AlH, RH, WM-L, and JS planned and performed the experiments, RL, OS, and BS designed the study and planned and supervised the research project. AK performed microbiome analysis. KK and AnH wrote the manuscript. AS and NW provided genetically engineered MAdCAM-KO mice. RL and OS supervised the research and edited the manuscript. All authors read and approved the final manuscript.

### Conflict of Interest Statement

OS serves on the editorial boards of the Multiple Sclerosis Journal, and Therapeutic Advances in Neurological Disorders and has served on data monitoring committees for Pfizer and TG Therapeutics without monetary compensation. OS has advised EMD Serono and Genzyme and currently receives grant support from Sanofi Genzyme. OS received travel support from Shire. RL received compensation for activities with Bayer, Biogen, Genzyme, Merck, Novartis, Roche, and TEVA as well as research support from Biogen and Novartis. The remaining authors declare that the research was conducted in the absence of any commercial or financial relationships that could be construed as a potential conflict of interest.

## References

[1] Butcher EC Picker LJ. Lymphocyte homing and homeostasis. Science. (1996) 272:60–7. 10.1126/science.272.5258.60 8600538

[2] Hamann A Andrew DP Jablonski-Westrich D Holzmann B Butcher EC. Role of alpha 4-integrins in lymphocyte homing to mucosal tissues in vivo. J Immunol. (1994) 152:3282–93. 7511642

[3] Nakache M Lakey Berg E Streeter PR Butcher EC. The mucosal vascular addressin is a tissue-specific endothelial cell adhesion molecule for circulating lymphocytes. Nature. (1989) 337:179–81. 10.1038/337179a0 2911352

[4] Kraal G Schornagel K Streeter PR Holzmann B Butcher EC. Expression of the mucosal vascular addressin, MAdCAM-1, on sinus-lining cells in the spleen. Am J Pathol. (1995) 147:763–71. 7677187 PMC1870972

[5] Schippers A Leuker C Pabst O Kochut A Prochnow B Gruber AD. Mucosal Addressin cell-adhesion molecule-1 controls plasma-cell migration and function in the small intestine of mice. Gastroenterology. (2009) 137:924–33. 10.1053/j.gastro.2009.05.039 19450594

[6] Clahsen T Pabst O Tenbrock K Schippers A Wagner N. Localization of dendritic cells in the gut epithelium requires MAdCAM-1. Clin Immunol. (2015) 156:74–84. 10.1016/j.clim.2014.11.005 25464027

[7] Cannella B Cross AH Raine CS. Relapsing autoimmune demyelination: a role for vascular addressins. J Neuroimmunol. (1991) 35:295–300. 1955571 10.1016/0165-5728(91)90183-8

[8] McDonald SA Palmen MJ Van Rees EP MacDonald TT. Characterization of the mucosal cell-mediated immune response in IL-2 knockout mice before and after the onset of colitis. Immunology. (1997) 91:73–80. 9203968 10.1046/j.1365-2567.1997.00217.x PMC1364037

[9] Kanwar JR Harrison JE Wang D Leung E Mueller W Wagner N. β7 integrins contribute to demyelinating disease of the central nervous system. J Neuroimmunol. (2000) 103:146–52. 10.1016/S0165-5728(99)00245-3 10696909

[10] Kanwar JR Kanwar RK Wang D Krissansen GW. Prevention of a chronic progressive form of experimental autoimmune encephalomyelitis by an antibody against mucosal addressin cell adhesion molecule-1, given early in the course of disease progression. Immunol Cell Biol. (2000) 78:641–5. 10.1046/j.1440-1711.2000.00947.x 11114975

[11] Kato S Hokari R Matsuzaki K Iwai A Kawaguchi A Nagao S. Amelioration of murine experimental colitis by inhibition of mucosal addressin cell adhesion molecule-1. J Pharmacol Exp Ther. (2000) 295:183–9. 10991977

[12] Matsuzaki K Tsuzuki Y Matsunaga H Inoue T Miyazaki J Hokari R. *In vivo* demonstration of T lymphocyte migration and amelioration of ileitis in intestinal mucosa of SAMP1/Yit mice by the inhibition of MAdCAM-1. Clin Exp Immunol. (2005) 140:22–31. 10.1111/j.1365-2249.2005.02742.x 15762871 PMC1809333

[13] Farkas S Hornung M Sattler C Edtinger K Steinbauer M Anthuber M. Blocking MAdCAM-1 *in vivo* reduces leukocyte extravasation and reverses chronic inflammation in experimental colitis. Int J Colorectal Dis. (2006) 21:71–8. 10.1007/s00384-004-0709-y 15856265

[14] Kamada N Seo SU Chen GY Núñez G. Role of the gut microbiota in immunity and inflammatory disease. Nat Rev Immunol. (2013) 13:321–35. 10.1038/nri3430 23618829

[15] Haghikia A Jörg S Duscha A Berg J Manzel A Waschbisch A. Dietary fatty acids directly impact central nervous system autoimmunity via the small intestine. Immunity. (2015) 43:817–29. 10.1016/j.immuni.2015.09.007 26488817

[16] Hammer A Yang G Friedrich J Kovacs A Lee DH Grave K. Role of the receptor Mas in macrophage-mediated inflammation *in vivo*. Proc Natl Acad Sci USA. (2016) 113:14109–14. 10.1073/pnas.1612668113 27872279 PMC5150410

[17] Rivera-Nieves J Olson T Bamias G Bruce A Solga M Knight RF L-selectin, α4β1, and α4β7 integrins participate in CD4^+^ T cell recruitment to chronically inflamed small intestine. J Immunol. (2005) 174:2343–52. 10.1038/mi.2014.22 15699171

[18] Viney JL Jones S Chiu HH Lagrimas B Renz ME Presta LG. Mucosal addressin cell adhesion molecule-1: a structural and functional analysis demarcates the integrin binding motif. J Immunol. (1996) 157:2488–97. 8805649

[19] Connor Elaine M Eppihimer Michael J Zenichi M Neil GD Grisham Matthew B Expression of mucosal addressin cell adhesion molecule-1 (MAdCAM-1) in acute and chronic inflammation. J Leukoc Biol. (1999) 65:349–55. 10.1002/jlb.65.3.349 10080539

[20] O'Neill JK Butter C Baker D Gschmeissner SE Kraal G Butcher EC. Expression of vascular addressins and ICAM-1 by endothelial cells in the spinal cord during chronic relapsing experimental allergic encephalomyelitis in the Biozzi AB/H mouse. Immunology. (1991) 72:520–5. 1674735 PMC1384371

[21] Leung E Greene J Ni J Raymond L Lehnert K Langley R. Cloning of the mucosal addressin MAdCAM-1 from human brain: identification of novel alternatively spliced transcripts. Immunol Cell Biol. (1996) 74:490–6. 10.1038/icb.1996.81 8989586

[22] Briskin M Winsor-Hines D Shyjan A Cochran N Bloom S Wilson J. Human mucosal addressin cell adhesion molecule-1 is preferentially expressed in intestinal tract and associated lymphoid tissue. Am J Pathol. (1997) 151:97–110. 9212736 PMC1857942

[23] Pullen N Molloy E Carter D Syntin P Clemo F Finco-Kent D. Pharmacological characterization of PF-00547659, an anti-human MAdCAM monoclonal antibody. Br J Pharmacol. (2009) 157:281–93. 10.1111/j.1476-5381.2009.00137.x 19366349 PMC2697799

[24] Allavena R Noy S Andrews M Pullen N CNS elevation of vascular and not mucosal addressin cell adhesion molecules in patients with multiple sclerosis. Am J Pathol. (2010) 176:556–62. 10.2353/ajpath.2010.090437 20035048 PMC2808064

[25] Döring A Pfeiffer F Meier M Dehouck B Tauber S Deutsch U TET inducible expression of the α4β7-integrin ligand MAdCAM-1 on the blood–brain barrier does not influence the immunopathogenesis of experimental autoimmune encephalomyelitis. Eur J Immunol. (2011) 41:813–21. 10.1002/eji.201040912 21341265

[26] Yednock TA Cannon C Fritz LC Sanchez-Madrid F Steinman L Karin N. Prevention of experimental autoimmune encephalomyelitis by antibodies against α4βl integrin. Nature. (1992) 356:63–6. 10.1038/356063a0 1538783

[27] Steffen BJ Butcher EC Engelhardt B. Evidence for involvement of ICAM-1 and VCAM-1 in lymphocyte interaction with endothelium in experimental autoimmune encephalomyelitis in the central nervous system in the SJL/J mouse. Am J Pathol. (1994) 145:189–201. 7518194 PMC1887301

[28] Engelhardt B Laschinger M Schulz M Samulowitz U Vestweber D Hoch G The development of experimental autoimmune encephalomyelitis in the mouse requires alpha4-integrin but not alpha4beta7-integrin. J Clin Invest. (1998) 102:2096–105. 10.1172/JCI4271 9854045 PMC509164

[29] Bullard DC Hu X Schoeb TR Collins RG Beaudet AL Barnum SR. Intercellular adhesion molecule-1 expression is required on multiple cell types for the development of experimental autoimmune encephalomyelitis. J Immunol. (2007) 178:851–7. 10.4049/jimmunol.178.2.851 17202346

[30] Mueller C Macpherson AJ. Layers of mutualism with commensal bacteria protect us from intestinal inflammation. Gut. (2006) 55:276–84. 10.1136/gut.2004.054098 16407387 PMC1856516

[31] Yacyshyn B Meddings J Sadowski D Bowen-Yacyshyn MB. Multiple sclerosis patients have peripheral blood CD45RO+ B cells and increased intestinal permeability. Dig Dis Sci. (1996) 41:2493–8. 9011463 10.1007/BF02100148

[32] Gupta G Gelfand JM Lewis JD. Increased risk for demyelinating diseases in patients with inflammatory bowel disease. Gastroenterology. (2005) 129:819–26. 10.1053/j.gastro.2005.06.022 16143121

[33] Flügel A Berkowicz T Ritter T Labeur M Jenne DE Li Z. Migratory activity and functional changes of green fluorescent effector cells before and during experimental autoimmune encephalomyelitis. Immunity. (2001) 14:547–60. 10.1016/S1074-7613(01)00143-1 11371357

[34] Odoardi F Sie C Streyl K Ulaganathan VK Schläger C Lodygin D. T cells become licensed in the lung to enter the central nervous system. Nature. (2012) 488:675–9. 10.1038/nature11337 22914092

[35] Ivanov II Frutos R de L Manel N Yoshinaga K Rifkin DB Sartor RB. Specific microbiota direct the differentiation of IL-17-producing T-helper cells in the mucosa of the small intestine. Cell Host Microbe. (2008) 4:337–49. 10.1016/j.chom.2008.09.009 18854238 PMC2597589

[36] Lathrop SK Bloom SM Rao SM Nutsch K Lio CW Santacruz N. Peripheral education of the immune system by colonic commensal microbiota. Nature. (2011) 478:250–4. 10.1038/nature10434 21937990 PMC3192908

